# mRNA and Next-Generation Vaccine Platforms for Pandemic Influenza Preparedness

**DOI:** 10.3390/vaccines14030247

**Published:** 2026-03-07

**Authors:** Rick A. Bright

**Affiliations:** Bright Global Health, Waterford, VA 20197, USA; rbright@bglobalhealth.org

**Keywords:** pandemic influenza, mRNA vaccines, vaccine platforms, pandemic preparedness, influenza vaccine manufacturing, 100 Days Mission, global health security

## Abstract

Pandemic influenza remains a persistent global threat with the potential to cause widespread morbidity, mortality, and economic disruption. Despite decades of preparedness efforts, current influenza vaccine systems remain constrained by long production timelines, early strain-selection requirements, and limited flexibility once a pandemic is underway. The COVID-19 pandemic fundamentally reshaped expectations for vaccine development and deployment, demonstrating that platform-based technologies, particularly messenger RNA (mRNA) vaccines, can deliver safe and effective vaccines at unprecedented speed when supported by regulatory readiness, manufacturing capacity, and coordinated public–private investment. Drawing on lessons from COVID-19, recent Phase III clinical trial data for seasonal influenza mRNA vaccines, and global preparedness initiatives such as the 100 Days Mission, this expert perspective examines how mRNA and other next-generation vaccine technologies could strengthen preparedness for a future influenza pandemic. It reviews evidence related to platform speed, clinical performance, manufacturing adaptability, regulatory pathways, and global access, while also highlighting emerging scientific frontiers, including artificial intelligence–augmented immunogen design and innovations in vaccine delivery. It argues that sustained investment in adaptable vaccine platforms, coupled with advances in delivery, manufacturing, and data-driven design, will be critical to improving global readiness and reducing the impact of the next influenza pandemic.

## 1. Introduction: The Persistent Threat of Pandemic Influenza

Pandemic influenza remains one of the most credible and persistent biological threats to global health, economic stability, and national security [1,2]. Novel influenza viruses continue to emerge from animal reservoirs, evolve rapidly, and spread efficiently in human populations. Recent analyses emphasize that the underlying drivers of influenza pandemic risk, including global travel, dense human–animal interfaces, and viral genetic plasticity, have intensified rather than diminished in recent years [1,2,3].

Despite sustained global investment in influenza surveillance and vaccine programs, preparedness gaps remain [4]. Seasonal influenza vaccines provide meaningful protection against morbidity and mortality in routine years, but the systems used to produce and deploy them are poorly suited for pandemic response. Most licensed influenza vaccines rely on manufacturing paradigms that require early strain selection, months-long production timelines, and limited capacity to adapt once manufacturing has begun [5,6]. When novel strains emerge or antigenic drift accelerates, these constraints can result in delayed availability, strain mismatch, and reduced population-level impact [6,7].

The COVID-19 pandemic marked a turning point in how rapidly vaccines can be developed and deployed during a global health emergency. Platform-based technologies, particularly mRNA vaccines, demonstrated that vaccines could be designed, manufactured, clinically evaluated, and distributed within months rather than years, while still meeting rigorous standards for safety, efficacy, and regulatory oversight [8,9,10,11], an achievement reflected in recent analyses of COVID-19 vaccine development timelines and technologies. These experiences reshaped expectations for pandemic preparedness and informed the articulation of the 100 Days Mission, which seeks to enable the delivery of safe and effective vaccines for emergency use within approximately 100 days of identifying a pandemic threat [12,13,14].

Influenza presents a particularly stringent test of these ambitions. Unlike SARS-CoV-2, influenza viruses undergo continual antigenic drift and may reassort rapidly after emergence, complicating vaccine design and increasing the risk of mismatch [6,7]. Achieving meaningful impact against pandemic influenza therefore requires not only speed, but adaptability, manufacturing resilience, and the ability to revise vaccine composition as new data emerge.

This perspective advances a central argument: pandemic influenza preparedness must evolve from a strain-forecasting model anchored in early antigen selection and fixed manufacturing processes to a platform-adaptive model capable of late-stage antigen revision, distributed manufacturing, and iterative update. The distinction is not merely technological but structural. Preparedness should be evaluated not only by installed production capacity, but by the capacity to adapt composition and scale in real time as viral evolution unfolds.

In this framework, mRNA and other next-generation vaccine technologies are not presented as replacements for traditional platforms, but as enabling components of a more adaptive preparedness architecture. By integrating advances in platform design, regulatory science, manufacturing strategy, and immunologic understanding, pandemic response systems can shift from static production models toward dynamic, revision-capable vaccine ecosystems better aligned with the biological realities of influenza.

## 2. Lessons from COVID-19 Relevant to Pandemic Influenza

The COVID-19 pandemic demonstrated that vaccine development timelines are increasingly determined by platform readiness and system preparedness rather than scientific feasibility alone. Rapid genomic sequencing, early sharing of viral data, and advances in platform technologies enabled vaccine candidates to enter clinical trials within weeks of pathogen identification [8,9,10,11]. Equally important were regulatory innovations, including rolling data submissions, early engagement with regulators, and parallel manufacturing scale-up conducted at financial risk [9,10,11].

These approaches allowed development, evaluation, and production activities to proceed simultaneously, compressing timelines without abandoning evidentiary standards. Large Phase III clinical trials were completed, extensive post-authorization safety monitoring systems were deployed, and manufacturing quality requirements were maintained throughout [8,9,10,11,15,16]. This experience directly challenges the notion that speed and rigor are inherently incompatible.

For influenza, similar approaches could offer substantial benefits. Traditional influenza vaccine development requires strain-selection decisions many months in advance, based on incomplete forecasts of viral evolution [7]. Once manufacturing begins, opportunities to adjust vaccine composition are extremely limited. Platform-based systems could allow strain-selection decisions to be made later, informed by more complete epidemiologic and virologic data, and potentially updated as conditions evolve.

The 100 Days Mission formalizes these lessons by setting a concrete preparedness target [12,13,14]. Achieving this goal requires more than platform technologies alone. It depends on sustained warm-base manufacturing capacity, secure and diversified supply chains, trained workforces, regulatory familiarity with platform approaches, and pre-established clinical trial and surveillance networks that can be activated immediately [12,13,14]. COVID-19 demonstrated that these enabling systems must be maintained between crises if rapid response timelines are to be realistic rather than aspirational.

## 3. mRNA Vaccines and Influenza Pandemic Preparedness

Influenza viruses present a compelling use case for mRNA vaccine platforms. Rapid mutation, antigenic drift, and reassortment challenge conventional vaccine approaches that require long lead times and fixed production processes [6,7]. Pandemic strains emerging from avian or other animal reservoirs may evolve substantially in the early phases of spread, increasing the likelihood that early vaccine formulations will become mismatched.

Recent clinical evidence supports the feasibility of mRNA vaccines for influenza. Large Phase III trials of seasonal influenza mRNA vaccines have demonstrated robust immunogenicity and favorable safety profiles in older adults, a population at heightened risk for severe influenza outcomes [17,18]. These trials establish regulatory familiarity with the platform for influenza indications and demonstrate that late-stage evaluation is achievable at scale.

These data have important implications for pandemic preparedness. Demonstration of clinical performance and acceptable safety profiles in seasonal settings provides a foundation for accelerated development in a pandemic context. In contrast to egg-based or other long-lead manufacturing systems, mRNA platforms allow antigen sequences to be updated rapidly without fundamentally altering the production process [10,11]. This capability could enable vaccine composition decisions to be revisited later in the course of a pandemic, rather than being locked in months in advance.

The relevance of this flexibility is illustrated by recent severe seasonal influenza outbreaks dominated by A(H3N2) viruses, where partial mismatch between vaccine and circulating strains has been observed [6,19]. In such settings, a platform that allows rapid reformulation and redeployment could reduce the consequences of mismatch and improve population-level protection, even if updates occur after the initial wave of transmission.

Manufacturing considerations further distinguish mRNA vaccines from traditional influenza vaccines. mRNA production relies on standardized, cell-free processes that can be scaled using modular facilities [10,11]. While not eliminating supply-chain dependencies, this approach reduces reliance on biological substrates and allows production systems to remain largely unchanged when antigen sequences are updated. When combined with sustained warm-base capacity, these attributes support faster scale-up and greater responsiveness during emergencies [12,13,14]. Key platform attributes are summarized in Table 1.

## 4. Traditional and Emerging Influenza Vaccine Technologies

While mRNA platforms offer distinct advantages in speed and adaptability, traditional egg-based and cell-based influenza vaccines remain foundational to global pandemic preparedness. Pandemic preparedness should not rely on a single technological solution. A diversified portfolio of vaccine approaches reduces risk and increases the likelihood that effective countermeasures can be deployed across a range of scenarios.

Established influenza vaccine platforms, including egg-based, cell-culture, and recombinant protein vaccines, continue to play an important role in pandemic preparedness. These technologies benefit from decades of regulatory experience, large installed manufacturing capacity, and advance purchase agreements that support baseline supply in many countries. While their longer production timelines and limited flexibility constrain rapid adaptation, they remain essential components of a diversified preparedness portfolio, particularly for early supply and global coverage.

Recent analyses estimate that global seasonal influenza vaccine manufacturing capacity has remained relatively stable since 2019 at roughly 1.5 billion doses annually, with more than 80% of supply relying on egg-based production. Even with this installed base, historical experience suggests that conventional approaches are unlikely to provide rapid, equitable global coverage early in a pandemic. During the 2009 H1N1 pandemic, the vaccine was first available roughly four months after the WHO pandemic declaration and became widely available closer to six months, after substantial transmission had already occurred [20,21,22]. By the six-month point, global production reached roughly 534 million pandemic vaccine doses, well below the aspirational goal of sufficient doses for two billion people [23].

Recombinant protein vaccines offer advantages related to stability and established manufacturing pathways and have demonstrated feasibility for influenza [24]. Nanoparticle-based vaccine designs seek to enhance antigen presentation and may support broader immune responses by displaying conserved influenza epitopes in optimized configurations [24]. Self-amplifying RNA vaccines aim to retain the adaptability of mRNA while reducing dose requirements, potentially improving manufacturing efficiency and expanding supply [25].

Advances in protein expression platforms also remain highly relevant. Innovations in microbial, plant-based, and cell-free expression systems seek to lower costs, accelerate scale-up, and expand geographic manufacturing capacity [24]. These approaches complement mRNA platforms by broadening the technological base available for pandemic response.

Global preparedness efforts increasingly emphasize portfolio approaches rather than single-platform bets. CEPI’s Disease X and pandemic vaccine investments reflect this strategy, supporting multiple vaccine modalities in parallel to increase the probability that at least one approach can be rapidly and effectively deployed when a pandemic strain emerges [14]. Publicly available CEPI portfolio data further illustrate how platform technologies, including mRNA, are being advanced alongside protein, viral vector, and other approaches to hedge scientific and manufacturing risk, including under CEPI’s “Disease X” framing [26].

Taken together, traditional and emerging influenza vaccine technologies represent complementary components of a resilient pandemic preparedness portfolio, each contributing distinct strengths across speed, scale, adaptability, and global access.

## 5. Manufacturing, Supply Chains, and Global Readiness

Manufacturing capacity and supply-chain resilience ultimately determine whether vaccine innovation translates into public-health impact. Traditional influenza vaccine production is constrained by long lead times, centralized facilities, and biological dependencies that limit surge capacity during emergencies [5,6].

Platform-based manufacturing models offer an alternative, but only if supported by sustained readiness. The concept of a warm base refers to maintaining facilities, workforces, quality systems, and supplier relationships in an operational state between emergencies [12,13,14]. For mRNA vaccines, this includes validated production lines, fill-finish capacity, access to specialized raw materials, and regulatory familiarity with platform processes.

Analyses from global preparedness initiatives emphasize that achieving compressed response timelines requires advance commitments to at-risk manufacturing and contractual mechanisms that can be activated immediately when a threat is identified [12,13,14]. Without these enabling systems, even highly adaptable platforms risk reverting to crisis-driven delays.

Global readiness also requires expanded manufacturing capacity beyond a small number of high-income countries. Efforts to build regional manufacturing capability aim to reduce dependence on limited suppliers and improve access during pandemics [27,28]. While technology transfer alone is insufficient, sustained investment in training, regulatory capacity, and supply networks can enhance resilience and equity.

Access to mRNA vaccines in low- and middle-income countries remains constrained by several factors, including higher current production costs, cold-chain requirements, limited regional manufacturing, and the need for specialized technical and regulatory expertise. These barriers underscore the importance of sustained investment in technology transfer, workforce development, regulatory strengthening, and supply-chain localization, rather than serving as arguments against continued platform development. WHO’s mRNA Technology Transfer Programme and related manufacturing investments are designed to address these barriers by building regional know-how and production capacity, but they require sustained funding and coordination to translate platform potential into equitable access [20,27,28,,].

Although mRNA vaccines have higher per-dose costs today compared with some legacy influenza vaccines, cost trajectories continue to decline as manufacturing scales and processes mature. In the context of a pandemic, the value of rapid deployment, reduced morbidity and mortality, and avoided economic disruption must be considered alongside unit price. Advance purchase agreements with existing influenza vaccine manufacturers remain a critical preparedness tool and should be complemented, not displaced, by investments in adaptable platforms capable of responding to evolving threats. Experience from 2009 suggests that when supply is constrained early, advance purchase agreements can reinforce inequities by prioritizing deliveries to high-income buyers unless paired with mechanisms that protect access for low- and middle-income countries [29].

## 6. Gaps, Risks, and Scientific Frontiers

Despite their promise, mRNA vaccines and other next-generation platforms face unresolved scientific and operational challenges. Correlates of protection for influenza remain incompletely defined, complicating rapid optimization and comparison across platforms. Durable protection is particularly important in pandemics, where multiple waves may occur and booster strategies must be planned.

Influenza presents immunologic complexities that differ materially from other respiratory viruses and that directly influence vaccine strategy. Antigenic drift can occur not only seasonally but during the early phases of a pandemic, particularly as population immunity begins to exert selective pressure on emerging strains [6,7]. During the 2009 H1N1 pandemic, genetic diversification occurred within months of global spread, illustrating how viral evolution can complicate early vaccine strain selection. In such settings, manufacturing paradigms that require fixed composition decisions months in advance are inherently vulnerable to mismatch.

Immune imprinting, sometimes described as “original antigenic sin,” further shapes pandemic responses. Prior exposure to seasonal influenza strains influences B-cell memory and antibody repertoires, potentially biasing immune responses toward conserved epitopes encountered earlier in life rather than novel antigenic features of a pandemic strain [6]. This effect may partially explain age-stratified differences in pandemic outcomes and vaccine effectiveness. Understanding how imprinting interacts with novel platforms, including mRNA-based antigen expression and multivalent or nanoparticle display strategies, remains an active area of investigation.

Correlates of protection for influenza are also more complex than for many other viral pathogens. While hemagglutination inhibition (HAI) titers have long served as regulatory benchmarks serving as a surrogate marker for immunity, they do not fully capture protection mediated by neuraminidase-directed antibodies, Fc-mediated effector functions, or T-cell responses that may contribute to cross-strain protection [3,6]. In addition, mucosal IgA responses in the respiratory tract may contribute to protection against infection and transmission, yet these are not routinely measured in standard regulatory immunogenicity endpoints [30]. Pandemic scenarios may therefore require broader immunologic endpoints, particularly if antigenic drift reduces the predictive value of strain-specific neutralizing antibodies. Advances in systems immunology and high-dimensional immune profiling, combined with computational modeling of viral evolution [31,32,33], offer opportunities to refine correlates and guide antigen design. Efforts to develop broadly protective or “universal” influenza vaccines targeting conserved regions of hemagglutinin, including stalk-based immunogens, illustrate the importance of immune breadth beyond strain-specific neutralization and further highlight limitations of single-epitope correlates [34].

Durability of protection adds another layer of complexity. Pandemic waves may unfold over extended periods, and waning immunity could coincide with viral evolution. Platforms that allow rapid reformulation and regulatory pathways for strain updates may enable iterative optimization rather than one-time deployment. In this context, adaptability is not simply a matter of speed; it is a mechanism for maintaining relevance as both virus and host immunity evolve.

Together, these influenza-specific immunologic realities reinforce the argument that pandemic preparedness must prioritize adaptive capacity. The objective is not merely accelerated first-dose availability, but the ability to revise, optimize, and redeploy vaccines in response to dynamic viral and immunologic landscapes.

Advances in artificial intelligence and computational biology offer new opportunities to address these challenges. Machine-learning approaches are increasingly used to analyze large influenza sequence datasets, identify conserved epitopes, predict antigenic drift, and guide immunogen design [31,32,33]. When paired with mRNA platforms, computationally designed immunogens could enable faster updates or reduce the need for frequent reformulation by prioritizing targets less susceptible to drift.

Innovations in vaccine delivery may further amplify the impact of rapid platforms. Oral recombinant vaccines offer simplified administration and the potential to induce mucosal immunity [30,35]. Self-administered vaccine microarray patches could increase coverage during both routine immunization and emergency campaigns [36]. Recent advances have demonstrated thermostable mRNA vaccines produced via microneedle printing, reducing reliance on cold-chain infrastructure and enabling more decentralized deployment [37].

Maintaining public trust remains essential. Transparent safety monitoring, clear communication, and consistent regulatory standards are critical to ensuring that accelerated development does not undermine confidence. Large-scale pharmacovigilance systems deployed during COVID-19 demonstrated that rare adverse events can be detected and evaluated rapidly in real-world settings [15,16]. These systems must remain integral to pandemic influenza preparedness.

## 7. Conclusions

Pandemic influenza preparedness requires more than incremental improvements to existing vaccine systems. The biological realities of influenza, including antigenic drift, immune imprinting, evolving correlates of protection, and the potential for multi-wave global spread, expose structural limitations in manufacturing models anchored to early strain selection and fixed production cycles. Preparedness must therefore be evaluated not solely by installed manufacturing capacity, but by adaptive capacity: the ability to update composition, scale production, and deploy vaccines in response to real-time epidemiologic and immunologic data.

The experience of COVID-19 demonstrated that platform-based technologies can compress development timelines dramatically when supported by regulatory readiness, sustained manufacturing infrastructure, and coordinated public–private investment. For influenza, the value of such platforms extends beyond speed alone. Adaptability, including the potential for later strain selection, iterative reformulation, and integration of improved immunologic insights, may prove equally critical.

Four strategic priorities emerge from this analysis.

First, sustained warm-base manufacturing must be maintained across both traditional and platform-based technologies. Diversified capacity reduces risk and ensures that surge production can begin immediately when a pandemic threat is identified.

Second, regulatory pathways for strain updates and platform-based modifications should be further clarified and harmonized internationally. Pre-established playbooks for composition updates would allow adaptive reformulation without restarting full evidentiary processes.

Third, regional manufacturing capacity and technical expertise must expand, particularly in low- and middle-income countries. Investments in technology transfer, workforce development, and regulatory strengthening are essential to ensure that adaptive platforms translate into equitable access rather than concentrated supply.

Fourth, continued investment in influenza immunology, including refinement of correlates of protection, characterization of immune imprinting effects, and development of broadly protective immunogen strategies, will be necessary to fully leverage adaptable platforms.

Pandemic influenza will not wait for ideal conditions. A preparedness architecture that integrates traditional manufacturing strengths with adaptable platform technologies offers the most resilient path forward. The question is no longer whether rapid vaccine development is scientifically feasible, but whether global systems are structured to sustain adaptive readiness between crises.

## Figures and Tables

**Table 1:** Comparison of Influenza Vaccine Technologies for Pandemic Preparedness.

Vaccine Technology	Development Speed	Manufacturing Flexibility	Late Strain Update Possible	Supply Chain Complexity	Regulatory Familiarity	Pandemic Readiness Profile	Representative Examples
Egg-based inactivated (standard dose)	Slow (months)	Low	No	High (egg supply dependent)	High	Large installed global capacity; limited adaptability	Fluzone; Fluarix
Egg-based inactivated (high-dose/adjuvanted)	Slow (months)	Low	No	High	High	Enhanced immunogenicity for older adults; same manufacturing constraints	Fluzone High-Dose; Fluad
Cell-based inactivated	Moderate	Low–Moderate	No	Moderate	High	Reduced egg adaptation risk; moderate flexibility	Flucelvax
Recombinant protein	Moderate	Moderate	Limited	Moderate	Moderate	Avoids egg adaptation; scalable but not fully platform-based	Flublok
Live attenuated influenza vaccine (LAIV)	Moderate	Low	No	High	High	Intranasal delivery; mucosal immunity; temperature-sensitive constraints	FluMist
Viral vector (non-LAIV recombinant vectors)	Moderate	Moderate	Limited	Moderate	Moderate	Platform potential; limited influenza licensure	Candidates in development
mRNA	Fast (weeks)	High (platform-based)	Yes	Lower, standardized	Increasing	High adaptability; modular manufacturing; regulatory familiarity expanding	mRNA-1010 (Moderna, Phase 3); Pfizer candidate (Phase 3)
Self-amplifying RNA	Fast	High	Yes	Lower (emerging)	Limited–Moderate	Dose-sparing potential; platform adaptability; early clinical stage	Multiple candidates in early clinical trials

Abbreviations: mRNA, messenger RNA.

## Data Availability

No new data were created or analyzed in this study. Data sharing is not applicable.
